# A Case Report of Paralysis and Respiratory Difficulty in a Patient With a Recent History of Complicated Pregnancy - An Uncommon Expression of Distal Renal Tubular Acidosis

**DOI:** 10.7759/cureus.50630

**Published:** 2023-12-16

**Authors:** Pirya Kumari, FNU Jitidhar, FNU Kiran, Ameet Kumar, Deepak Kumar

**Affiliations:** 1 Medicine, Peoples University of Medical and Health Sciences For Women, Karachi, PAK; 2 Internal Medicine, One Brooklyn Health Interfaith Medical center, Brooklyn, USA; 3 Pathology, University of Missouri School of Medicine, Columbia, USA; 4 Medicine, Saint Vincent Hospital, Erie, USA; 5 Pathology, University of Missouri, Columbia, USA

**Keywords:** sjögren’s syndrome, pregnancy, peripheral muscle weakness, respiratory muscle weakness, distal renal tubular acidosis

## Abstract

Renal tubular acidosis type 1 (RTA-1) is a disorder where kidneys are unable to acidify urine, which ultimately results in normal anion gap metabolic acidosis. Its initial presentations and subsequent clinical manifestations can vary depending on the underlying cause and severity of the disease. We report a case of a 26-year-old female with a recent history of complicated pregnancy. She presented to a tertiary care hospital with quadriplegia and shortness of breath and required ventilator support. The extensive workup revealed that the patient had RTA-1 in association with Sjögren's syndrome. There are only a few cases of RTA-1 reported where the diagnosis was made during the pregnancy. By reporting this case of RTA-1 with rare initial clinical presentation and a recent complicated pregnancy, we propose that further research studies should be carried out in this area to explore a possible statistically significant association between pregnancy (and its complications) and RTA-1 exacerbation.

## Introduction

Renal tubular acidosis (RTA) is one of the few causes of metabolic acidosis with normal serum anion gaps. RTA is mainly of three types: proximal, distal, and hyperkalemic RTA [1]. Distal renal tubular acidosis (RTA-1) can be hereditary due to a genetic mutation of enzymes and/or channels in the distal tubule and collecting ducts, but it can also be secondary to some systemic diseases, including but not limited to Sjögren's syndrome [2]. Distal renal tubular acidosis has variable clinical presentations and is often associated with hypokalemia. It rarely manifests into quadriplegia and respiratory failure. Furthermore, pregnancy has been reported to exacerbate RTA-1 due to its physiologic changes and volume overload on the kidneys [3].

Here, we present a case of a female patient with undiagnosed RTA-1 that was associated with Sjögren's syndrome. The patient was admitted for quadriplegia and eventually went into respiratory failure. She had a recent history of complicated pregnancy.

## Case presentation

A 26-year-old female patient, mother of two children, with a history of cesarean section (c-section) three months ago, was admitted to medical ICU with complaints of all four-limb weakness for three days and shortness of breath for one day. The weakness started in the lower limbs and progressed rapidly to involve her upper limbs. Over two days, it worsened to the point that she was unable to move her fingers. She became short of breath, and ultimately required ventilator support. She also had a history of vomiting for seven months. The frequency of episodes of vomiting decreased from thrice a week to once a week after her c-section three months ago. The associated symptom was the loss of appetite for the last seven months. Besides the above findings, a review of the other systems was unremarkable. Her obstetric history included two c-sections. She had two sons, both delivered through c-section. Her elder son was born at term and was healthy, while her second pregnancy was complicated, resulting in a preterm delivery at 28 weeks; the baby, however, survived without complications. She was not taking any medications except for multivitamins and, as needed, antiemetics. Her family history was non-contributory. Her parents and siblings were alive and healthy. She neither had exposure to pets such as dogs, cats, or birds nor had she a history of recent travel, history of swimming, tick bite, or hiking. She did not have any fever, neck rigidity, diplopia, joint problems, photosensitivity, alopecia, or other signs or symptoms to suggest a possible underlying autoimmune or infectious process.

On examination, a thin, lean lady in respiratory distress was lying in bed. Her respiratory rate was 35 breaths per minute. Her blood pressure was 100/60 mmHg, and pulse rate was 125 beats per minute. She was conscious and oriented. Her limbs were flaccid with the power of 0/5 in all four limbs. Deep tendon reflexes were absent, but her peripheral sensations were intact. The chest was clear with vesicular breathing bilaterally, and the abdominal examination was benign. Laboratory investigations of the patient are given below (Table 1).

**Table 1:** Laboratory investigations

Blood			
Parameter	Result	Unit	Reference range
Hemoglobin	9.1	g/dL	11.5 - 15
Platelets	150	10^9/L	150 - 400
Total leukocyte count	21	10^9/L	4 - 10
Neutrophils	80%	N/A	40 - 75%
Lymphocytes	11%	N/A	20 - 45%
pH (arterial blood)	7.09	N/A	7.3 - 7.4
Pco2 (arterial blood)	40	N/A	40 - 44
Po2 (arterial blood)	76	mmHg	75 - 100
Oxygen saturation (arterial blood)	88.6%	N/A	94 - 100%
Bicarbonate (arterial blood)	15.1	mmol/L	22 - 28
Serum sodium	150	M Eq/L	136 - 149
Serum calcium	8.1	mg/dL	8.6 - 10.5
Serum potassium	1.8	M Eq/L	3.8 - 5.2
Serum bicarbonate	16	M Eq/L	25 - 29
Serum Phosphorus	1.52	mg/dL	2.7 - 4.5
Serum chloride	120	M Eq/L	98 - 107
Serum magnesium	2.22	mg/dL	1.6 - 2.6
Serum urea	50	mg/dL	10 - 50
Serum creatinine	0.67	mg/dL	0.6 - 0.9
Serum albumin	3.13	g/dL	3.6 - 49
Serum C-reactive protein	5.47	mg/dL	0 - 0.5
Erythrocyte sedimentation rate	45	mm/1^st^ hour	0 - 20
Prothrombin time	10.9	Seconds	11.4
Serum procalcitonin	0.175	ng/mL	<0.5
Serum C3	1.4	g/L	0.5 - 1.5
Serum C4	0.4	g/L	0.1 - 0.4
Plasma parathyroid hormone (intact)	71	pg/ml	16 - 87
Serum 25-hydroxy vitamin D	8.9	ng/ml	30 - 150
Blood culture	No bacterial growth		
Urine			
pH	6.5	N/A	
Potassium	17	M Eq/L	20 - 67
Sodium	98	M Eq/L	54 - 150
Calcium	6.9	mg/dL	
Chloride	108	M Eq/L	46 - 168
Urine culture	No bacterial growth		
Autoimmune Profile			
Anti-nuclear antibodies	Negative		
Anti-mitochondrial antibodies	Negative		
SS-A/Ro antibodies	>70	U/ml	<3.2
SS-B/La antibodies	1.5	U/ml	<8.0
U1-RNP antibodies	0.8	U/ml	<3.2
Sm-antibodies	0.45	U/ml	<3.2
Scl-70 antibodies	0.30	U/ml	<3.2
Anti-dsDNA (IgG)	2.6	U/ml	<20

Since SS-A/Ro antibodies were strongly positive, we, therefore, performed Schirmer's test in a search to detect underlying Sjögren's syndrome as a cause of RTA-1, and it resulted positive. Further workup that included thyroid profile, liver function tests, and cerebrospinal fluid analysis came out normal. ECG did not reveal any abnormality except for sinus tachycardia.

## Discussion

Distal renal tubular acidosis is a disorder of hyperchloremic normal anion gap metabolic acidosis. It causes a spectrum of clinical presentations depending upon the cause and severity of the disease. Severe RTA-1 with genetic mutation can develop early in infancy or childhood, while the mild form can manifest in adolescence. Whereas acquired RTA-1 secondary to autoimmune disorders, e.g. Sjögren's syndrome, is more commonly observed in adulthood [1].

RTA-1 is diagnosed in a patient when urinary pH is alkaline, i.e., urine pH>5.5 in a context of existing metabolic acidosis or an induced acidosis [2,4]. Urine anion gap (UAG) is performed to differentiate the renal or extrarenal cause of hyperchloremic metabolic acidosis. UAG remains positive in RTA-1 and negative if normal anion gap metabolic acidosis is due to extrarenal etiology, e.g., diarrhea [1,2]. Moreover, UAG becomes negative in proximal renal tubular acidosis (RTA-2) provided serum HCO3- is low.

Acquired RTA-1 is associated with Sjögren's syndrome in 5-25% of cases [5]. Patients with Sjögren's syndrome present due to glandular and/or extraglandular involvement of the disease. RTA-1 is one of its extraglandular expressions. One large Indian study shows that only 8.1% of patients come to clinical attention by themselves due to their subjective complaint of sicca symptoms [6]. Sjögren's syndrome diagnosis is mostly made based on criteria set by the American European Consensus Group (AECG) [6,7].

Hypokalemia is found in patients with ailments of RTA-1. One proposed theory behind it is that since hydrogen is not excreted, potassium is wasted in order to maintain electroneutrality in urine, which results in low serum potassium [5]. Caruana and Buckalew, in their study, demonstrated that 28% of patients with low serum potassium are associated with RTA-1 [4]. Hypokalemia can cause symptoms of polyuria and polydipsia, but quadriplegia with impending respiratory failure is a rare occurrence [8-10]. One Asian study shows that 5.4% of patients with Sjögren's syndrome present with hypokalemic paralysis as their first manifestation [6]. In quite a few instances, undiagnosed asymptomatic RTA-1 is later uncovered and reported during pregnancy due to hypokalemia and associated complications [11,12]. It can be speculated from these cases that physiologic changes in pregnancy might have incited otherwise occult RTA-1.

Our patient had a chronic history of nausea and vomiting starting in the second trimester of pregnancy. Her basic workup did not show any abnormality; however, she continued to have her problem. Her pregnancy was terminated, and the baby was delivered via c-section with a possible diagnosis of hyperemesis gravidarum. After delivery, she continued to have emesis, but the frequency decreased from before. Three months post c-section, she started developing weakness in the lower limbs, which progressed rapidly and involved the upper limbs. With a history of weakness and paralysis for three days, she was admitted to our intensive care unit, where she became severely short of breath and was put on a ventilator. After an extensive workup, she was diagnosed with hypokalemic paralysis with RTA-1 secondary to Sjögren's syndrome and was treated with alkali therapy and potassium supplements along with steroids. She improved and was discharged home on oral supplements.

We believe that it is possible that recurrent vomiting in our patient during pregnancy could have been due to hyperemesis gravidarum since she started getting better post c-section. Her emesis after delivery was possibly secondary to RTA-1, which might have become apparent due to a complicated pregnancy and/or subsequent surgery.

## Conclusions

We suggest that any female patient with muscular weakness or paralysis and respiratory insufficiency during or after pregnancy should be investigated for possible RTA-1 and associated electrolyte imbalances. Once diagnosed, appropriate urgent steps should be taken to prevent devastating consequences. Furthermore, research studies are needed to understand the possible impact of pregnancy and its complications on RTA-1 exacerbation.

## References

[1] Yaxley J, Pirrone C (2016). Review of the diagnostic evaluation of renal tubular acidosis. Ochsner J.

[2] Batlle D, Haque SK (2012). Genetic causes and mechanisms of distal renal tubular acidosis. Nephrol Dial Transplant.

[3] Alkhasoneh M, Jacobs J, Kaur G (2019). A case of severe metabolic acidosis during pregnancy. Clin Case Rep.

[4] Caruana RJ, Buckalew VM Jr (1988). The syndrome of distal (type 1) renal tubular acidosis. Clinical and laboratory findings in 58 cases. Medicine (Baltimore).

[5] Both T, Zietse R, Hoorn EJ, van Hagen PM, Dalm VA, van Laar JA, van Daele PL (2014). Everything you need to know about distal renal tubular acidosis in autoimmune disease. Rheumatol Int.

[6] Sandhya P, Jeyaseelan L, Scofield RH, Danda D (2015). Clinical characteristics and outcome of primary Sjogren’s syndrome: a large Asian Indian cohort. Open Rheumatol J.

[7] Ram R, Swarnalatha G, Dakshinamurty KV (2014). Renal tubular acidosis in Sjögren's syndrome: a case series. Am J Nephrol.

[8] Fujimoto T, Shiiki H, Takahi Y, Dohi K (2001). Primary Sjögren's syndrome presenting as hypokalaemic periodic paralysis and respiratory arrest. Clin Rheumatol.

[9] Poux JM, Peyronnet P, Le Meur Y, Favereau JP, Charmes JP, Leroux-Robert C (1992). Hypokalemic quadriplegia and respiratory arrest revealing primary Sjögren's syndrome. Clin Nephrol.

[10] Gombar S, Mathew PJ, Gombar KK, D'Cruz S, Goyal G (2005). Acute respiratory failure due to hypokalaemic muscular paralysis from renal tubular acidosis. Anaesth Intensive Care.

[11] Cheng CY, Nair GV, Salmon Y (1983). Renal tubular acidosis presenting in pregnancy with severe hypokalaemia. Aust N Z J Obstet Gynaecol.

[12] Carminati G, Chena A, Orlando JM, Russo S, Salomón S, Carena JA (2001). Distal renal tubular acidosis with rhabdomyolysis as the presenting form in 4 pregnant women (Article in Spanish). Nefrologia.

